# Effects of the modified “Yijing Fang” on semen quality and reproductive hormones in male yaks with kidney yang deficiency: a focus on mitochondrial apoptosis pathways

**DOI:** 10.1590/1984-3143-AR2024-0139

**Published:** 2025-05-26

**Authors:** Panfeng Chang, Hua Wang

**Affiliations:** 1 Animal Science Research Laboratory, Gansu Animal Science and Veterinary Medicine Institute, Ping Liang, Gansu, China

**Keywords:** yaks, modified “Yijing Fang”, mitochondrial apoptosis pathways, oligoasthenozoospermia, kidney yang deficiency

## Abstract

Intensive semen collection often leads to “kidney yang deficiency” in male yaks, with symptoms like lethargy and poor semen quality. Our modified “Yijing Fang” was tested. Eight 3 - 6-year-old male yaks with this condition were split into treatment and negative control groups; four healthy yaks were a blank control. The treated yaks got the formula in feed for 5 days, then a 20-day break, repeating 3 times over 65 days. Semen was collected, and cAMP, cGMP, T/E2 levels, and mitochondrial apoptosis factors were analyzed. Results showed the formula boosted semen quality. cAMP and testosterone rose (p < 0.01), cGMP and estradiol fell (p < 0.01), cAMP/cGMP and T/E2 ratios climbed (p < 0.01). Bcl-2 upregulated, Bax, Cyt-C, and Caspase-3 downregulated (p < 0.01). In conclusion, the modified “Yijing Fang” is effective for yak reproduction, enhancing hormone secretion and reducing sperm apoptosis. Long-term studies are needed.

## Introduction

Yaks (Bos grunniens) are critical livestock native to the Tibetan Plateau and adjacent high-altitude regions of China (Ayalew et al., 2021), playing a vital role in maintaining ecosystem stability, ensuring food security, promoting socio-economic development, and preserving cultural traditions (Jing et al., 2022). Currently, the global yak population is approximately 17.6 million, with around 17 million being domesticated and a few thousand remaining wild (Jing et al., 2022). Over 30 local quality breeds and two artificially cultivated varieties have been identified (Wang et al., 2019). Yaks exhibit remarkable adaptability to challenging high-altitude environments characterized by low oxygen levels, high barometric pressure, and prolonged food scarcity (Lan et al., 2016; Niayale et al., 2021). This unique adaptability relates closely to their distinctive morphology, physiology, and traits shaped by extensive natural and artificial selection (Lan et al., 2018; Qiu et al., 2012).

However, the increasing adoption of intensive yak farming practices, particularly artificial insemination, significantly impacts the health of male yaks. The demands of high-pressure and low-oxygen conditions during semen collection and breeding impair their physiological condition, leading to symptoms of diminished health, such as lethargy, poor semen quality, and weakened immune function, resembling “kidney yang deficiency.” This deterioration may further contribute to reproductive disorders, including oligospermia and asthenozoospermia.

Traditional Chinese medicine, with over two millennia of history, recognizes herbal medicine as a critical green feed additive. Classical texts, such as “Shennong's Classic of Materia Medica” and “Bencao Gangmu,” guide the development of modern veterinary practices. As a vital aspect of traditional Chinese medicine, veterinary herbal medicine is aligned with China's internationalization efforts, contributing significantly to animal disease prevention and sustainable livestock breeding. Following the comprehensive ban on antibiotics in animal feed in 2020, the use of veterinary herbal medicine has surged. Since then, the Ministry of Agriculture and Rural Affairs has approved numerous traditional veterinary medicines that integrate traditional and modern medicine, replace antibiotics, and promote environmentally friendly practices.

In light of this, the current study aims to investigate the effects of a modified “Yijing Fang” on the semen quality, reproductive hormones, and mitochondrial apoptosis in male yaks. The objective is to enhance semen quality and reproductive performance, while also providing a viable alternative to hormonal treatments. This research seeks to provide experimental evidence for improving reproductive performance in male livestock and a scientific basis for optimizing the production of the modified “Yijing Fang,” ensuring the sustainable development of yak breeding.

## Methods

### Experimental animals

This experiment was conducted at the Zongxing Cattle and Sheep Breeding Cooperative located in Qinghai Province. A total of male yaks aged between 3 and 6 years were selected based on clinical symptoms indicative of kidney yang deficiency, which included unkempt fur, diminished libido, weakness in the lumbar and pelvic regions, and increased duration spent lying down. All selected animals were free from infectious diseases, such as brucellosis. Following artificial semen collection, 5 μL of raw semen was subjected to examination under a 400× microscope. A diagnosis of kidney yang deficiency was made if at least one of the following criteria was met: (1) sperm density < 2 × 10^7/mL; (2) sperm motility < 50%; or (3) survival rate < 60%. Eight yaks diagnosed with kidney yang deficiency were randomly assigned to either a negative control group or an experimental group. Additionally, four yaks of comparable age and size exhibiting normal reproductive performance were selected as a positive control group.

### Experimental methods

#### Chinese herbal medicine materials

The modified “Yijing Fang” was derived from the patent titled “A Chinese Herbal Prescription and Preparation Method for Improving Bull Semen Quality” (Patent No.: CN202110188011.7). Its composition included the following ingredients: 35 g of Epimedium, 35 g of Cuscuta seed, 35 g of Cynomorium, 30 g of Cistanche, 30 g of Rehmannia glutinosa, 30 g of Morinda officinalis, 20 g of Psoralea corylifolia, 30 g of Schisandra chinensis, 30 g of Cnidium monnieri, 30 g of Allium tuberosum, and 30 g of Dioscorea opposita. All herbs were procured from the Lanzhou Huanghe Herbal Medicine Market, ground into a coarse powder, and incorporated into the yak feed. Each yak received one dose daily for a treatment cycle of five days, followed by a 20-day break. This cycle was repeated three times, amounting to a total treatment duration of 65 days. During the study, yaks were provided with standard roughage feed and had free access to water.

#### Semen quality evaluation

The ethical approval number: Gansu Agricultural University, 2024-038, which has been approved by the Animal Medical Ethics Committee of Gansu Agricultural University.

Semen was collected from yaks utilizing the artificial vagina method. The volume of raw semen along with the duration for the bull to mount was documented. The color, odor, and pH of the collected semen were assessed. Any semen samples not conforming to the standards outlined in “Bovine Frozen Semen” (GB4143-2008) were discarded. Sperm density was measured in situ using a sperm density analyzer (PigDoc), where a 5 mL aliquot of raw semen was diluted 50-fold in physiological saline. A drop of this dilution was placed on a slide and covered with a coverslip. Sperm motility was evaluated at 35°C using a portable microscope (Beidoubo). The rate of sperm abnormalities was determined by preparing a smear of raw semen, fixing it with 95% alcohol, staining with 0.5% brilliant green, and examining 300 to 500 spermatozoa under a microscope at 400-600× magnification. Acrosome integrity was assessed by diluting the raw semen 50-fold in 3% physiological saline, drying the smear, and staining with Giemsa solution. Semen samples meeting the criteria established in “Bovine Frozen Semen” (GB4143-2008) were cryopreserved in liquid nitrogen to prevent agitation.

#### Yak serum collection

Blood samples were collected from the jugular vein of the aforementioned yaks with the assistance of an on-site veterinarian. The collected blood was allowed to clot at 4°C for 24 hours before separations, and serum was labeled and stored at -80°C for subsequent analysis.

#### Determination of cAMP/cGMP, T/E2 levels in yak serum

Reagents used included cAMP (BY-B90390, Xuanke, Shanghai), cGMP (BY-B90388, Xuanke, Shanghai), testosterone (T) (XK-SJH-1060, Xuanke, Shanghai), and estradiol (E2) (BKE7582). The procedure commenced with thawing the separated serum, standards, and reagents at 4°C. Prepared samples and standards were then added, followed by an incubation at 37°C for 30 minutes. After five washes, color reagents A and B were added, and the mixture was incubated at 37°C for an additional 10 minutes. A stop solution was added, and the optical density (OD) value was read within 15 minutes. The results were subsequently calculated.

#### Western Blot analysis of Bcl-2/Bax, Cyt-C, and Caspase 3 expression in yak semen

Frozen semen was thawed and centrifuged at 10,000 rpm for 5 minutes at 4°C; the supernatant was discarded. The pellet was washed with PBS and centrifuged at 10,000 rpm for another 5 minutes at 4°C. This washing step was repeated at least five times to eliminate the cryoprotectant. Proteins were then extracted using the RIPA tissue/cell lysis solution kit, with the supernatant constituting the semen protein. The proteins were separated by SDS-PAGE and transferred to a PVDF membrane (YA1701, Solarbio, Beijing). Membranes were blocked using 5% non-fat milk at 37°C for 2 hours and then incubated with primary antibodies Bcl-2 (1:1000), Bax (1:2000), Cyt-C (1:1000), Caspase 3 (1:1000), and GAPDH (1:2500, Abcam, USA) at 4°C for 12 hours. After washing with TBST for 1 hour, membranes were incubated with a secondary antibody (rabbit anti-mouse IgG, 1:5000, Abcam, USA) for 2 hours, followed by another wash of 1.5 hours. Detection was performed using ECL, and protein expression was quantified using ImageJ, with GAPDH serving as the internal control. All experiments were completed in triplicate.

#### Quantitative RT-PCR

Total RNA was extracted from all yak semen samples utilizing Trizol, followed by measurement of OD260/280 and concentration via spectrophotometry, and dilution to 300 ng/μL. Using the reverse transcription kit (AH411-02, Yinsengebio, Beijing), RNA was reverse-transcribed into cDNA, which was further diluted to 300 ng/μL. RT-PCR analysis was conducted using the LightCycler 96® system (Roche, Basel, Switzerland). All reactions were carried out in triplicate, and the cycle threshold (Ct) method was employed for quantification, with GAPDH as the internal control. Relevant primers were designed by Qingke Biotechnology Co., Ltd., with primer sequences detailed in Table 1.

**Table 1:** Primers sequence of factors related to mitochondrial apoptosis.

**Genes**	**Primers**
Bcl-2	F: 5’-GGTGGAGGAGTCCTTCAG-3’;
	R: 5’-GGTTGACGCTCTCCACAC-3’
Bax	F: 5’-CGAGTGGCGGCTGAAATGT-3’;
	R: 5’-GGCCTTGAGCACCAGTTTG-3’
Cyt-C	F: 5’CATACTGTGGAAAAGGGAGGC-3’;
	R: 5’-CAGGGATGTACTTCTTGGGAT-3’
Caspase 3	F: 5’-TGGCGAAATGCAAAGAACGG-3’;
	R: 5’-TGCATGAAAAGCAGAATCGGT-3’
GADPH	F:5’-GGTACCAGGGCTGCTTT-3’;
	R: 5’-CTGTGCCGTTGAACTTGC-3’

#### Statistics

All data were statistically analyzed using SPSS 26.0 software. One-way ANOVA was applied to compare the differences between groups, and results were expressed as mean ± standard deviation. Multiple comparisons were conducted using the Duncan test. Graphical representations were generated using GraphPad Prism 8.0 (GraphPad, USA), with p-values less than 0.05 considered statistically significant.

## Results

### Evaluation of yak semen quality

The semen samples obtained from all three groups of yaks exhibited a milky-white appearance accompanied by a slightly pungent odor. Notably, the control group, characterized by kidney-yang deficiency, exhibited a more translucent semen color, while the normal group displayed a slightly yellowish tint. A comprehensive summary of additional semen quality parameters for each group is provided in Table 2.

**Table 2:** Semen index of yak.

**Group**	**Semen volume(mL)**	**Sperm density (×10^8/mL)**	**Sperm motility (%)**	**Sperm abnormality rate (%)**	**Acrosome integrity (%)**	**PH**
Normal group	5.72±2.13	13.45±4.52^a^	73.44±9.11^a^	11.37±3.07^a^	87.28±6.54^a^	7.1
Control group	3.85±1.98	5.34±2.08^c^	59.27±8.56^c^	15.44±4.79^c^	69.06±2.34^c^	6.52
Modified “Yijing Fang” group	4.64±2.03	7.28±3.44^b^	67.83±5.41^b^	13.99±3.25^b^	75.45±7.32^b^	6.94

Note: Data in the same column with different superscript letters indicate statistically significant differences (P<0.05).

### Determination of cAMP/cGMP, T/E2 Levels in Yak Serum

**Figure 1:**
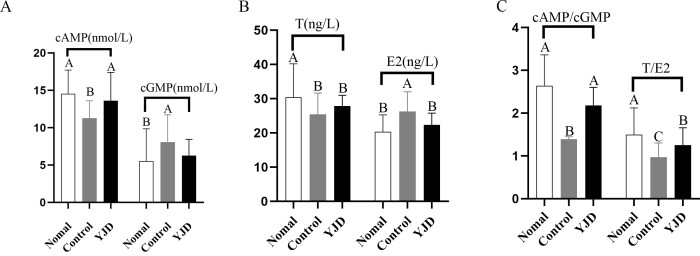
The Serum cAMP cGMP,T and E2 concentration was determined by ELISA. A:cAMP and cGMP; B:T and E2; C:cAMP/cGMP; T/E2 (Different capital letters: *P*<0.01)

As shown in Figure 1, both the control group and modified “Yijing Fang” group showed a significant decrease in serum cAMP concentration compared to the normal group. Specifically, serum cAMP levels in both the normal and the modified “Yijing Fang” groups were substantially higher than those in the control group (p < 0.01), while no significant difference was observed between the normal and modified “Yijing Fang” groups (p > 0.05). Conversely, cGMP concentration exhibited an inverse trend, with the normal group displaying significantly lower levels than the control group (p < 0.01). No substantial differences were noted among the normal, “Yijing Fang,” and control groups concerning cGMP levels (p > 0.05). Furthermore, the cAMP/cGMP ratio was reduced in both the control and modified “Yijing Fang” groups compared to the normal group, with significant differences confirmed (p < 0.01).

In terms of serum testosterone (T) concentration, the trends mirrored those observed for cAMP. The control group and modified “Yijing Fang” group demonstrated lower T levels relative to the normal group, with both groups significantly higher than the control group (p < 0.01), while no significant difference was identified between the normal and modified “Yijing Fang” groups (p > 0.05). In contrast, the estradiol (E2) concentration exhibited a trend opposite to that of T, with both the normal and modified “Yijing Fang” groups registering significantly lower E2 levels than the control group (p < 0.01). No significant differences were observed between the normal and the modified “Yijing Fang” groups (p > 0.05). The T/E2 ratio, however, was significantly elevated in the normal group compared to both the modified “Yijing Fang” and control groups (p < 0.01); additionally, modified “Yijing Fang” group had a significantly higher ratio than the control group (p < 0.01).

**Figure 2:**
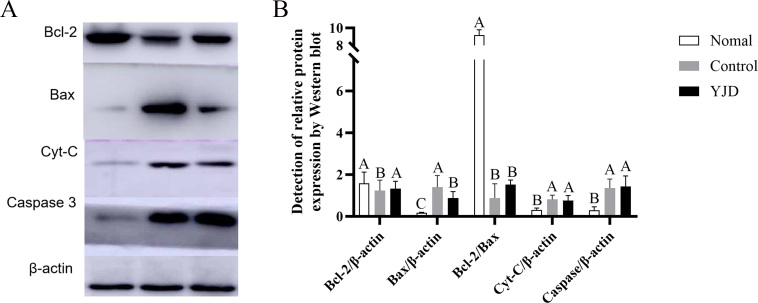
Effect of modified YLD on expression of mitochondrial apoptosis factor protein in testis of kidney-yang deficiency yaks; A. Detection of mitochondrial apoptosis-related factors by Western blot; B. Detection of reative protein expression by Western blot. (Different capital letters: P<0.01).

The Western blot results shown in Figure 2 revealed the following: Bcl-2: The expression levels in the normal and modified “Yijing Fang” groups were significantly higher compared to the control group (p < 0.01), with no significant differences between the normal and modified “Yijing Fang” groups (p > 0.05); Bax: The control group exhibited significantly higher expression compared to both the normal and modified “Yijing Fang” groups (P < 0.01), and the modified “Yijing Fang” group showed significantly higher levels than the normal group (P < 0.01); Cyt-C: The normal group had significantly lower expression levels compared to both the control and modified “Yijing Fang” groups (p < 0.01), with no significant differences observed between the modified “Yijing Fang” and control groups (p > 0.05); Caspase 3: Similar to Cyt-C, expression levels in the normal group were significantly lower than in the control and modified “Yijing Fang” groups (P < 0.01), while there were no significant differences between the modified “Yijing Fang” and control groups (P > 0.05); Bcl-2/Bax Ratio: The modified “Yijing Fang” groups at high, medium, and low doses all showed significantly higher ratios compared to all other groups (P < 0.01).

### Effects of the modified “Yijing Fang” on mRNA Expression of Mitochondrial Apoptosis-Related Factors in Semen from Kidney-Yang Deficient Yaks

**Figure 3:**
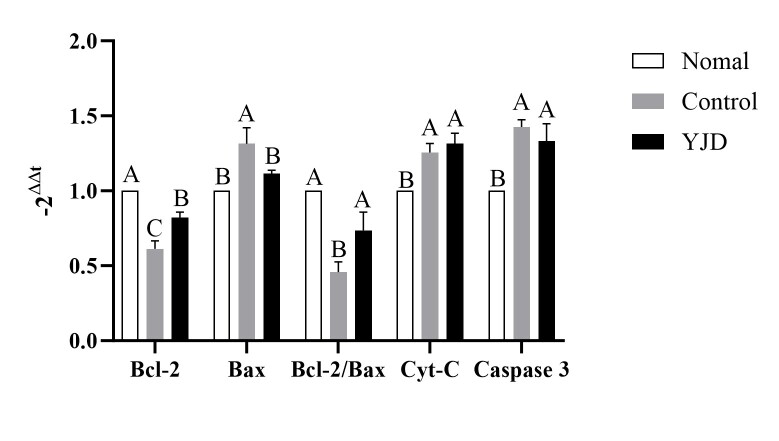
Effect of modified YLD on mRNA of mitochondrial apoptosis factors in semen of kidney-yang deficiency yaks (Different capital letters: p<0.01).

The outcomes of the quantitative RT-PCR analysis corroborate the findings presented in Section 2.3 and are detailed in Figure 3. For the Bcl-2 factor, the normal group showed significantly higher mRNA expression compared to both the modified “Yijing Fang” and control groups; in turn, the modified “Yijing Fang” group exhibited markedly higher levels than the control group (p < 0.01). For the Bax factor, the control group demonstrated the highest expression levels, whereas the normal group displayed significantly lower levels in comparison to the modified “Yijing Fang” group. Furthermore, the modified “Yijing Fang” group had significantly reduced expression relative to the control group (p < 0.01). In terms of Cyt-C and Caspase 3 factors, both the control and modified “Yijing Fang” groups exhibited significantly elevated levels compared to the normal group (p < 0.01), while no significant difference was found between the modified “Yijing Fang” and control groups (p > 0.05).

## Discussion

Traditional Chinese herbs such as Epimedium, Cuscuta, Cynomorium, and Lycium barbarum have long served as foundational formulas for tonifying Yin and enhancing Yang, with their efficacy recorded in historical pharmacopoeias for thousands of years. “Kidney essence deficiency and insufficient energy and blood” is a condition often seen in male cattle that undergo prolonged labor and breeding activities. According to the treatment principle of “tonifying the deficient,” the focus of therapy should center around warming and tonifying kidney Yang, nourishing Yin, generating fluids, and replenishing Qi and blood (Yánez-Ortiz et al., 2022). In this study, the evaluation standard for fresh bull semen indicated that semen quality is considered acceptable when sperm motility exceeds 65%, sperm density is ≥6 × 10^8 cells/ml, and sperm malformation rate is ≤15%. The control group's semen quality—reflected by sperm motility, density, and malformation rate—contradicted the reference standards for “Bull Frozen Semen,” indicating that the semen from Kidney-Yang deficient yaks does not meet the requirements for artificial insemination. Poor-quality semen can increase the failure rates of artificial insemination and lead to weak offspring that are susceptible to congenital diseases (Kowalczyk et al., 2022). Incorporating the modified “Yijing Fang” as a feed additive in the diet of Kidney-Yang deficient yaks considerably enhanced their semen quality. The findings align with those of Xue (Xue et al., 2022) and Tu (Tu et al., 2023), who reported that herbal formulas such as Epimedium, Cynomorium, and others can effectively enhance the quality of semen in male animals. Additionally, the active components of traditional Chinese medicine are widely utilized in the preservation of frozen semen. For example, compounds like hypericin and icariin have demonstrated the ability to effectively protect frozen sperm from oxidative stress and facilitate the removal of reactive oxygen species (ROS) post-thaw, thereby improving sperm vitality (Zhang et al., 2021). Semen consists of both sperm and seminal plasma, with the volume of semen primarily contingent upon the seminal plasma—secreted by accessory glands. Inflammatory conditions affecting the seminal vesicles, prostate, or bulbourethral glands can lead to reduced ejaculate volume. Nutraceuticals such as Dioscorea and Angelica, which act as tonifying herbs, may improve microcirculation and enhance glandular secretion. Moreover, tonifying herbs such as Morinda officinalis and Cuscuta have shown partial efficacy against accessory gland inflammation. The improved “Yijing Fang,” which synergistically combines Cuscuta, Dioscorea, and Morinda, helps enhance microcirculatory perfusion in accessory glands and prevent inflammation.

The concentrations and ratios of cAMP, cGMP, T, and E2 are established objective indicators for determining Kidney-Yang deficiency (Wang et al., 2023). Cyclic nucleotides cAMP and cGMP are ubiquitous second messengers that regulate various functions across nearly all eukaryotic cells. Their intracellular effects are mediated through discrete subcellular signaling microdomains (Sprenger and Nikolaev, 2013). Research indicates that Yang deficiency can lead to diminished adrenal system function and hormone secretion, resulting in a decrease in cAMP levels. cAMP mainly participates in the hormone synthesis and secretion processes of the pituitary gland, Leydig cells, and gonads by activating the protein kinase A (PKA) pathway; while cGMP influences sex hormones through interactions with the cAMP pathway (Chen et al., 2022). In this study, the application of the modified “Yijing Fang” on Kidney-Yang deficient yaks revealed its potential to elevate cAMP levels while concurrently decreasing cGMP, thereby regulating the balance of Yin and Yang in these yaks and offering therapeutic benefits for deficiency conditions. T and E2 are key hormones directly influencing reproductive organs (Shi et al., 2023). Elevated E2 levels have been identified as an indicator of Yang deficiency in male animals (Seong et al., 2023). Current medical research indicates that high E2 levels in male animals can hinder spermatogenesis, potentially resulting in oligospermia or infertility (Zhu et al., 2022b). Our findings demonstrated that E2 levels in the control group were significantly higher than in the blank group, correlating with poor semen quality, suggesting that the yaks in the control group may already be experiencing oligospermia. If intensified semen collection continues, it could further jeopardize fertility. In Chapter 3, the results from rat experiments indicated that the intervention with the modified “Yijing Fang” significantly ameliorated the morphology of damaged testes. This suggests that the formula can enhance the secretion of reproductive hormones such as T and E2 in Kidney-Yang deficient yaks, potentially providing therapeutic effects against oligospermia and promoting spermatogenesis. Moreover, preliminary research utilizing HPLC-MS qualitatively and quantitatively analyzed the formula's active components, which possess antioxidant, nitroprotective, anti-inflammatory, and anti-apoptotic effects, including quercetin, catechin, luteolin, rhamnetin, and icariin, confirming their therapeutic potential against oligospermia in Kidney-Yang deficient yaks (Chang et al., 2023; Liu et al., 2022; Wang et al., 2024; Wu et al., 2022).

Bcl-2 and Bax are key target molecules in endogenous apoptosis (Ostapchenko et al., 2019).Studies have shown that yaks exhibit higher expression of the Bcl-2/Bax ratio in the HPG axis when compared to beef cattle, suggesting a physiological adaptation to high-pressure, low-oxygen environments (Qin et al., 2021). Research by Wang Shulin et al. (Kamińska et al., 2024) indicated that in male infertile patients, Bcl-2 expression positively correlates with semen volume, concentration, progressive motility, and sperm viability. In contrast, Zhu et al. (2022a) found that in patients with oligospermia, the ratios of Bax/Bcl-2 and Caspase 3 were significantly lower than in normal males, suggesting that high sperm apoptosis rates may contribute to infertility. Our findings align with these observations, showing that Kidney-Yang deficient yaks exhibited significantly lower Bcl-2/Bax expression, sperm volume, and sperm density compared to healthy yaks, while Caspase 3 expression was conversely elevated. Cao Wei's findings suggest that “Yijing Fang” can improve oligospermia in mice through the ROS/MAPK-mitochondrial pathway, primarily by reducing ROS levels and inhibiting Cyt C release (Sun et al., 2022). In our study, the modified “Yijing Fang” also effectively lowered Cyt C expression, subsequently inhibiting sperm cell apoptosis. While the original formula including Epimedium, Cuscuta, and Angelica has shown pronounced effects, it remains at the experimental stage in rodent models and has not yet been patented for broader use. The modified “Yijing Fang” has been patented and combines Epimedium, Cuscuta, and Cynomorium with additional herbs like Rehmannia, Dioscorea, Morinda, and Fructus Psoraleae to enhance its efficacy. Furthermore, previous studies indicated a notable effect of this formula on melatonin levels in bull semen (Hua et al., 2021), suggesting that melatonin may enhance spermatogenesis by improving the hypothalamic-pituitary-gonadal axis and clear free radicals in bulls while regulating the expression of various antioxidant and pro-oxidant enzymes (Paiva et al., 2024; Zhao et al., 2023). Through our investigation of mitochondrial apoptosis-related factors in semen, we concluded that the modified “Yijing Fang” significantly enhances semen quality in Kidney-Yang deficient yaks, providing therapeutic effects against oligospermia.

## Conclusion

This study investigated the effects of modified “Yijing Fang” on semen quality and reproductive hormones in male yaks with kidney yang deficiency. Results indicate that the modified formula significantly enhances semen quality, as shown by increased sperm vitality, density, and motility. Treatment also elevated serum levels of cAMP and testosterone, while reducing cGMP and estradiol, thus improving the T/E2 ratio. The underlying mechanism appears to involve modulation of mitochondrial apoptosis pathways, evidenced by the upregulation of Bcl-2 and downregulation of Bax, Cyt-C, and Caspase-3. In summary, this study supports the efficacy of the modified “Yijing Fang” as an alternative to traditional hormonal treatments for enhancing reproductive performance in male yaks.

## Data Availability

No research data were used in this study.
